# Effect of Body Weight and Other Metabolic Factors on Risk of Non-Small Cell Lung Cancer among Veterans with HIV and a History of Smoking

**DOI:** 10.3390/cancers12123809

**Published:** 2020-12-17

**Authors:** Jose M. Garcia, Jennifer R. Kramer, Peter A. Richardson, Sarah Ahmed, Kathryn E. Royse, Donna L. White, Suchismita Raychaudhury, Elaine Chang, Christine M. Hartman, Michael J. Silverberg, Elizabeth Y. Chiao

**Affiliations:** 1 Geriatric Research, Education, and Clinical Center, VA Puget Sound HCS, Seattle, WA 98108, USA; jg77@uw.edu; 2 Department of Medicine, Division of Gerontology & Geriatric Medicine, University of Washington, Seattle, WA 98195, USA; 3 VA Health Services Research Center for Innovations in Quality, Effectiveness, and Safety (IQuESt), Michael E. DeBakey VA Medical Center, Houston, TX 77030, USA; jkramer@bcm.edu (J.R.K.); peterr@bcm.edu (P.A.R.); Sarah.Ahmed@bcm.edu (S.A.); Kathryn.E.Royse@kp.org (K.E.R.); dwhite1@bcm.edu (D.L.W.); Suchismita.Raychaudhury@bcm.edu (S.R.); drelainechang@gmail.com (E.C.); Christine.Hartman@va.gov (C.M.H.); 4 Texas Medical Center Digestive Diseases Center, Houston, TX 77030, USA; 5 Dan L. Duncan Cancer Center, Baylor College of Medicine, Houston, TX 77030, USA; 6 Department of Medicine, Baylor College of Medicine, Houston, TX 77030, USA; 7 Division of Research, Kaiser Permanente Northern California, Oakland, CA 94612, USA; michael.j.silverberg@kp.org

**Keywords:** AIDS, lung cancer, statins, cachexia, antiretroviral therapy

## Abstract

**Simple Summary:**

Among people living with HIV (PWH), there has been an increasing incidence of non-small cell lung cancer (NSCLC) and metabolic abnormalities, such as diabetes and high cholesterol, which affect the risk of NSCLC. In this article, we evaluate which metabolic risk factors increase the risk of NSCLC among PWH who smoke. Through a retrospective study that includes 33,351 veterans, we found that the risk of NSCLC was lower in well-controlled PWH (1.46 vs. 2.06/1000 patient/year [PY]). Metabolic factors associated with higher NSCLC risk included lower body weight at HIV diagnosis and a remote history of involuntary weight loss in PWH regardless of whether they had a well-controlled infection or not. Lower HDL and triglyceride levels increased the risk of NSCLC only in non-well-controlled smokers. Our results suggest these factors may be important to consider in targeting surveillance and for early identification of NSCLC in PWH smokers.

**Abstract:**

Among people living with HIV (PWH), there has been an increasing incidence of non-small cell lung cancer (NSCLC) and metabolic abnormalities, including dyslipidemia, which can modulate NSCLC risk. In this article, we evaluate which metabolic risk factors are associated with incident risk among PWH who smoke. This is done through a retrospective cohort study, using data of HIV+ veterans who smoke from the nationwide Veterans Affairs (VA) healthcare system. Data on diagnostic codes, medication, and laboratory values of 33,351 veterans were obtained using the VA’s Corporate Data Warehouse and Central Cancer Registry. We calculated NSCLC incidence and utilized Cox regression to determine metabolic factors associated with NSCLC risk. HIV+ cohort was 97.4% male; median age = 47 years and 20,050 (60.1%) well-controlled (≥80% follow-up time undetectable viral load). Crude incidence rates were lower in well-controlled PWH (1.46 vs. 2.06/1000 PY). Metabolic factors associated with incident NSCLC risk included lower BMI at HIV diagnosis and cachexia history in both groups, while HDL and triglycerides were significant in non-well-controlled smokers only. Our findings that lower BMI at HIV diagnosis, history of cachexia among individuals with well-controlled HIV, and cachexia presence at diagnosis are associated with increased risk of developing NSCLC in PWH with a history of smoking have important implications.

## 1. Introduction

With the development of active antiretroviral therapy (ART), leading to large numbers of HIV-positive individuals having a well-controlled infection, mortality in people living with HIV (PWH) has decreased dramatically [1]. However, there has been an increase in non-HIV related cancer that is a significant source of morbidity/mortality in this population. More people in the U.S. die from lung cancer than any other cancer [2], and PWH are at a higher risk of developing non-small cell lung cancer (NSCLC), the most common form of lung cancer, and have poorer outcomes [3]. Lung cancer represents 30% of all cancer deaths and 10% of all non-HIV related deaths in PWH [3].

PWH are also more likely to develop obesity and dyslipidemia, either as a result of the infection or as a side effect of ART [4]. In HIV-negative individuals, obesity is a significant risk factor for some cancers [5], and there is an inverse relationship between survival and a recent history of weight loss at NSCLC diagnosis [6]. Whether weight loss is a risk factor for developing lung cancer in PWH is unknown. Several other metabolic factors and medications are also thought to modulate lung cancer risk in HIV-negative individuals [7]. Diabetes may be associated with increased risk of certain cancers [8,9], with total and high-density lipoprotein (HDL) cholesterol inversely associated with lung cancer risk in some studies, but not others [10,11,12]; whereas, higher triglyceride levels have generally been associated with higher lung cancer risk [13]. In addition, statin use has not been shown to be associated with lung cancer in either PWH or HIV-negative individuals [14,15].

The extent to which HIV control, exposure to ART and other medications, or the presence of metabolic abnormalities modulate lung cancer risk in PWH is unknown. We, thus, evaluated the effect of body weight, history of weight loss, other metabolic factors, including diabetes status, HDL, LDL, and triglyceride levels, and exposure to medications to treat HIV and metabolic diseases on the incidence of NSCLC in a retrospective nationwide cohort study of veterans with HIV.

## 2. Results

**Figure 1:**
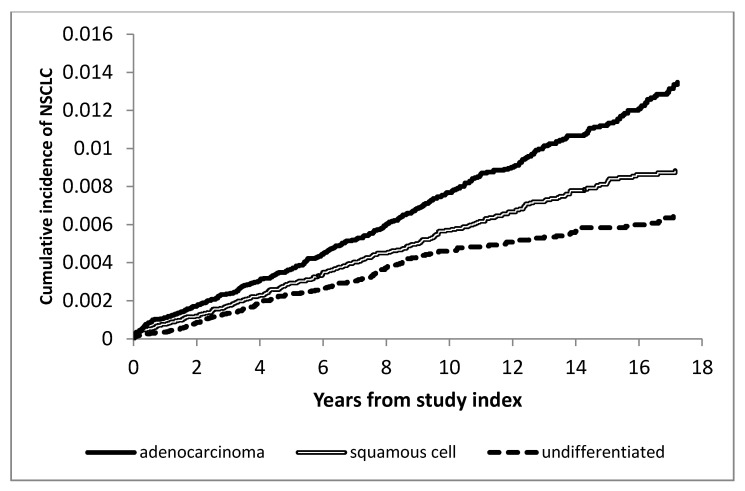
Cumulative incidence curve of non-small cell lung cancers by histology in people living with HIV (PWH) with a history of smoking.

We examined 46,662 patients with HIV at risk for NSCLC, of which 43,799 patients’ smoking status could be ascertained with 704 incident cases of NSCLC for a raw incidence rate of 1.485/1000 PY (95% CI: 1.378–1.599). Only 14 cases were ascertained among 9462 non-smokers, with an incidence rate of 0.147/1000 PY (95% CI: 0.080–0.246); the remaining 690 were among 33,351 smokers with an incidence rate of 2.700/1000 PY (95% CI: 1.920–3.692). Accordingly, the remaining analyses were restricted to “ever-smokers” (*n* = 33,351). By histopathology, the 690 incident NSCLC cases were divided into adenocarcinoma (*n* = 312; IR 0.824/1000 PY (95% CI: 0.736–0.921)), squamous cell (*n* = 217; IR 0.573/1000 PY (95% CI: 0.500–0.655)), and undifferentiated NSCLC (*n* = 161; IR 0.425/1000 PY (95% CI: 0.362–0.496)). Figure 1 and Figure S1, respectively, show the cumulative incidence of NSCLC by histology in PWH with a history of smoking and the overall incidence of NSCLC in PWH with a history of smoking.

Baseline characteristics at time of HIV diagnosis (index) for the complete smoking cohort and stratified by viral control are shown in Table 1. The subgroup with well-controlled infection were older (mean age in years: 48.6 ± 10.2 vs. 46.5 ± 10.3, *p*-value < 0.0001), more likely to be White (43.8% vs. 33.8%, *p*-value < 0.0001) and have a BMI > 30 (13.5% vs. 8.4%, *p*-value < 0.0001). Subjects with well-controlled infection were more likely to develop diabetes mellitus (5.9% vs. 5.1%, *p*-value < 0.0001), chronic obstructive pulmonary disease (COPD) (7.1% vs. 5.9%, *p*-value < 0.0001), and to receive statins before index date (7.5% vs. 4.9%, *p*-value < 0.0001) than those with poorly controlled HIV. Subjects with well-controlled infection were more likely to have nadir CD4 count > 500 (13.4% vs. 7.0%, *p*-value < 0.0001), >80% of follow-up time on ART (48.2% vs. 12.1%, *p*-value < 0.0001) and received a statin (50.6% vs. 31.4%, *p*-value < 0.0001) by end of follow-up than those with poorly controlled HIV. They were also less likely to have a history of alcohol (48.3% vs. 52.6%, *p*-value < 0.0001) or drug abuse (40.5% vs. 49.3%, *p*-value < 0.0001).

### 2.1. Risk Factors for NSCLC among Veterans with HIV

#### Univariate Analyses

Factors significantly associated with the risk of developing incident NSCLC in PWH with a history of smoking in the univariate analysis included: age, non-Hispanic ethnicity, HIV index date, current cachexia, low BMI, history of COPD, triglycerides <150 mg/dL, statin use, number of prior pneumonia episodes, and alcohol abuse [HR ranging from 6.01 (95% CI: 4.91–7.35) for current cachexia to 0.34 (95% CI: 0.19–0.58) for the Hispanic race] (Table 2). In the well-controlled subgroup, the pattern was similar except that the HIV index date showed no association with NSCLC, and alcohol use did not quite reach statistical significance. At the time of cancer diagnosis, there was no clear association between cancer stage and the rate of current cachexia for stages 2 or higher (stage I: 7.94%, stage II: 28.57%, stage III: 19.59%, and stage IV 25.75%); data not shown.

### 2.2. Multivariable Analyses

In the well-controlled subgroup, older age (60+ vs. <50 years HR = 3.79; 95% CI: 2.56–5.62), non-Hispanic ethnicity (Hispanic vs. white: HR = 0.35; 95% CI: 0.14–0.86), history of COPD (HR = 1.87; 95% CI: 1.42–2.47) and of prior pneumonia (HR = 1.64; 95% CI: 1.05–2.58), lower BMI at HIV diagnosis (BMI 30+ vs. 18.5–25 HR = 0.52; 95% CI: 0.31–0.86) and presence of current (HR = 6.34; 95% CI: 4.52–8.88) and distant cachexia (HR = 1.39; 95% CI: 1.01–1.93) were significant predictors of NSCLC in multivariable analysis (Table 3). In the not well-controlled, lower HDL and triglyceride levels were also predictors of NSCLC in addition to older age, non-Hispanic ethnicity, history of COPD and of multiple prior episodes of pneumonia, lower BMI and recent cachexia.

## 3. Discussion

In this large nationwide cohort study of veterans living with HIV who have a history of smoking, we found that metabolic factors, such as lower baseline BMI and recent cachexia are associated with higher NSCLC incidence in HIV-infected smokers regardless of the degree of viral control, even after adjusting for other immunologic variables (such as nadir CD4 count).

While overall survival among PWH has increased, the excess mortality associated with non-AIDS-defining malignancies is substantial [16]. The mechanisms underlying this increase are not understood well. Lung cancer is currently the most common non-AIDS-defining malignancy in PWH, and its incidence is three times higher than in the non-HIV infected [17]. PWH appear to develop lung cancer at a younger age [3], and these differences may not be entirely explained by other traditional factors, such as smoking [18]. The current retrospective study sheds light on potential metabolic risk factors that may influence NSCLC incidence in smokers living with HIV.

In HIV-negative individuals, obesity is known to increase the overall risk of cancer; however, the relationship between BMI and lung cancer is controversial. Calle et al. reported an inverse relationship between BMI and lung cancer, but a sub-analysis limited to non-smokers did not show this trend, suggesting it is mediated by smoking [5]. More recently, BMI was found to be inversely associated with the risk of lung cancer in current and former smokers [19]. In our PWH smoking subgroup, lower baseline BMI was one of the strongest predictors of subsequent NSCLC diagnosis. Moreover, this remained strongly significant even after adjusting by other potential confounders, such as age, a diagnosis of COPD, ethnicity, and recent cachexia. It was also interesting that this effect was seen regardless of HIV control and that it extended into the obese range (BMI > 30), which is usually considered unhealthy. A history of recent weight loss is often present at lung cancer diagnosis and is associated with poor prognosis in HIV-negative individuals [20]. We found that PWH with weight loss were 6–7 times more likely to have NSCLC if they had recent weight loss. Furthermore, in the well-controlled subgroup, we found that distant or prior weight loss was also independently associated with risk of NSCLC. These findings suggest that BMI and weight history should be taken into account when considering an individual’s risk for NSCLC and underscore the need for lung cancer screening in HIV-positive individuals with a history of smoking and low BMI or recent weight loss.

The association between HDL, LDL, and triglycerides and lung cancer in HIV-negative individuals is also controversial [10,11,12,13]. Given that lipid abnormalities are common in PWH, particularly in those receiving ART [21], we explored this association in our study. We found that lower levels of triglycerides and HDL were significantly associated with a higher risk of NSCLC, although this only reached significance in those whose HIV was not well-controlled. A recent meta-analysis on lipids and lung cancer in HIV-negative individuals suggests that high HDL and low triglycerides are protective [13]. Although these findings on HDL are similar to what we see in our cohort, the opposite association was seen for triglycerides in our study after controlling for other covariates that could modify this association, such as statin use, body weight, and recency of cachexia. Taken together, our data suggest an HIV-specific mechanism for this association that may be modulated by ART exposure, given that these agents are known to be associated with altered lipid levels [22,23]. More studies will be needed to establish the exact mechanisms and the potential causality of this association.

Interestingly, we did not find any impact of nadir CD4 count on the incidence of NSCLC among either the well-controlled or not well-controlled HIV subgroups. Thus, it is possible that other mediating variables associated with poor viral control (e.g., cases of pneumonias) may be driving the increased NSCLC incidence among people living with HIV rather than CD4 count in well-controlled individuals living with HIV.

This study has many strengths. It is the first study to our knowledge to report on the relative contribution of metabolic risk factors, including body weight, weight history, and lipid levels, to NSCLC risk in PWH. It involved a large nationwide cohort of PWH receiving care within the VA healthcare system, and thus, receiving largely comparable quality and quantity of care. Minorities were well represented, and our focus on Veterans is also an asset given that this is a population not always included in HIV research studies. Another is that veterans who use VA healthcare often receive most if not all of their care over an extended time period within this system, allowing for better longitudinal capture of outcomes than is possible in many other HIV cohorts that are often followed for a more limited time period or where not all data sources we had access to and leveraged in our analyses, including complete time-updating data on pharmacy and labs, as well as full capture of all BMIs from any clinical encounter for our extended study period. Also, the VA as national policy regularly updates risk factor screenings in all veterans, including for smoking and alcohol use, conveying advantage for the performance of large database research compared to use of Medicare or commercial insurance databases where this screening data is either not available or not systematically collected. Our use of a tiered approach to identify our HIV cohort using our validated algorithm, as well as the use of multiple methods to validly identify lung cancer, including using chart review and leveraging the VA’s adjudicated cancer registry, are other strengths of our study.

This study also has several limitations, including its retrospective nature that limits our ability to determine potential causation from the associations detected here. Also, the treatment of HIV is constantly evolving, and a temporal bias cannot be completely excluded. Further, our sample included mostly men given the demographic distribution of the veteran population, so these results may not be applicable to women. Finally, we were unable to ascertain details related to smoking history (e.g., pack-years), only the fact that individuals in the cohort were “ever” smokers. Thus, the “ever smoker” cohort may be somewhat heterogeneous with respect to their smoking history. However, the “ever smoker” cohort did have a significantly higher risk for NSCLC compared to the full cohort, and thus, allowed for stratification to decrease any residual confounding that smoking may have contributed to in multivariable modeling.

## 4. Materials and Methods

### 4.1. Methods

#### 4.1.1. Study Population and Design

We performed a retrospective cohort study using individual-level patient data from the national VA Corporate Data Warehouse (CDW) and the VA Central Cancer Registry (CCR). The CDW is a comprehensive automated VA database updated in near real-time and includes all inpatient/outpatient encounters, laboratory, pharmacy, and vital status information on all VA-users. The VA CCR is a national data repository for over 750,000 VA patients with cancer [24,25]. Cancer registrars at each VA site manually abstract data with quality assurance checks performed on aggregated data. It includes patient demographics, date of cancer diagnosis, primary site, histology, grade, tumor size, extension, staging, and treatment.

#### 4.1.2. HIV-Positive Cohort

Our nationwide HIV-positive cohort included patients aged ≥18 who fulfilled at least 2 of 3 criteria: (1) The laboratory criterion required ≥1 positive HIV antibody test by ELISA or Western Blot; be tested for HIV viral load (any +/−/indeterminate); or tested for CD4+ count; (2) the treatment criterion required at least one prescription in inpatient/outpatient pharmacy records for HIV antiretroviral therapy (ART); and (3) the diagnosis criterion required an inpatient or outpatient encounter with an International Classification of Diseases, Ninth Revision (ICD-9) code (042 or V08) or ICD-10 code (B20 and Z21) for HIV, as previously described [26]. We used the earliest date of the HIV diagnostic criteria as the index date for follow-up analyses unless it was before 1 October 1999 (inception date of most CDW data), in which case we assigned 1 October 1999 as the index date. The “well-controlled” subgroup was defined as veterans living with HIV who had evidence of utilizing ART with an undetectable HIV viral >80% of their individual follow-up time. We excluded veterans that were well-controlled in the absence of ART (*n* = 986) because we were unable to determine whether they were receiving ART outside the VA.

#### 4.1.3. Smoking Cohort

Evidence of a history of smoking was ascertained using comments from the CDW health factor questionnaire data [27] or the presence of smoking-related conditions as indicated by ICD codes and was categorized as “ever” vs. “never smoking” (ICD-9 codes, 305.1, 649.0, 989.84, V15.82; ICD-10 codes, F17.20, F17.21, Z72.0, T65.22, Z87.891, Z71.6) measured through the end of follow-up. Using the health factor data for smoking status has been validated by McGinnis et al., who reported substantial agreement with a kappa statistic of 0.72 when categories were collapsed into ever/never (sensitivity = 91%; specificity = 84%). To avoid confounding associated with smoking in the analysis, the main analyses were conducted in the “ever” smoking cohort.

#### 4.1.4. Variable Specification

##### Outcomes

We used a hierarchical approach to define the occurrence of NSCLC in our HIV-positive cohort. First, we identified all NSCLC in the gold-standard VA CCR based on primary site codes 162.X and C34 with histology codes for all NSCLC histological subtypes, including 800XX through 804XX, 807XX, 814XX, 824XX-848XX, as well as text searches. The VA Cancer Registry utilizes either ICD-O-2 or ICD-O-3 primary site and histology codes to identify tumors. Histology ICD-O codes are based on the NCI-funded Surveillance Epidemiology and End Results Program histology algorithms. Not all diagnoses were based on the 2015 WHO Classification, since most cases were from before 2015 by the nature of our cohort. Thus, the additional immunohistochemistry tools were not uniformly performed or reported for these cases. We further identified patients with any ICD-9 (162.X, 209.21) or ICD-10 code (C34, C7A.090) for malignant neoplasm of the lung from inpatient and outpatient encounter records in CDW. We then performed additional structured medical review under the direction of an oncologist to ascertain if patients who had only an ICD diagnostic code for NSCLC, but were not captured by the VA CCR had evidence supporting an NSCLC diagnosis. This hierarchical approach ensured the high validity of all captured NSCLC cases [28]. All-cause mortality was identified from the VA Vital Status file, which combines information from Medicare, VA, Social Security, and VA compensation and pension benefits to determine the date of death (sensitivity = 98.3%; specificity = 99.8% relative to National Death Index) [29].

##### Covariates

CDW data sources used included diagnoses and procedures from inpatient and outpatient encounters, laboratory results, and pharmacy dispensing records; these were used to ascertain baseline risk factors and comorbidities, as well as longitudinal time-updated risk factors.

##### Baseline Covariates

Demographics included age at study index, sex, and race/ethnicity (categorized into White, Black, Hispanic, other or unreported). Baseline BMI was defined as the closest BMI to the study index date, but limited to +/−1 year. The calendar year of HIV diagnosis was defined using the year of the earliest HIV diagnosis date in the VA from either the VA HIV Clinical Case Registry or VA CDW.

##### Time-Updated Covariates

Time-updated variables were evaluated at an interval of every 90 days. ICD-derived risk factors included the history of alcohol abuse (yes/no), substance abuse (non-cannabis), COPD, and pneumonia. For pneumonia, at each time point, a count was updated of previous episodes of pneumonia (in which these episodes were separated by at least a year of absence of pneumonia diagnosis codes). Diabetes was defined as the presence of ICD codes for diabetes (ICD-9 codes, 250.X; ICD-10 codes, E10.0–E14.9) or the first date of: Prescription for diabetes medication, high hemoglobin A1c (≥6.5%) or high blood glucose level (>11.1 mmol/L or >200 mg/dL); diabetes status was time-updated from this information.

The height/weight data were smoothed by cubic splines, allowing updating every 90 days. Cachexia was defined as a 5% drop in weight over a 6-month period (or 10% over a 12-month period). To track the further effects of episodes of cachexia, a time-updated cachexia scale was defined as (i) “none”: 0 at all times before the first episode of cachexia, (ii) “current cachexia”: 1 at the time of ascertainment of a cachexic episode, (iii) “recent cachexia”: After cachexia, an exponential decay term is applied every 90 days and (iv) “distant past or rebounded cachexia”: After a post-cachexia weight rebound, a further penalty term is applied to capture cachexic episodes prior to a year before censor or cancer.

Time-updated antiretroviral (ART) and statin use were examined as current use (yes/no) and % of relative follow-up time on drug (<50%, 50–80%, and >80%). Time-updated laboratory-based variables included lipids HDL (<40, 40–60, >60 mg/dL), LDL (<100, 100–129, 130–159, >160+ mg/dL), and triglycerides (<150, >150 mg/dL). In addition, time dependent nadir CD4 were derived from the longitudinal CD4 history, as was relative % time with undetectable HIV viral load from the viral load history. Because nadir CD4 and current CD4 were highly colinear, only nadir CD4 values were included.

##### Statistical Analysis

We examined the distributions of baseline patient characteristics by means with standard deviations, medians with interquartile ranges and density plots for continuous variables, and contingency tables for categorical variables. Since our objective was to examine incident cancer risk, we excluded patients with evidence of NSCLC prior to their index date. For patients with known HIV diagnoses before the year 2000 from HIV Clinical Case Registry, 1 October 1999 was designated as the index date because this is the inception date of the CDW data. Follow-up time at risk was calculated from index to the development of NSCLC, death, or 31 December 2016, whichever was earlier. We calculated incidence rates for NSCLC (with Poisson-based 95% confidence intervals) in our HIV positive smoking cohort overall and by smoking status per 1000 person/years at risk. Crude incidence was calculated accordingly for any NSCLC, as well as by specific histology (i.e., adenocarcinoma, squamous or undifferentiated). We also calculated these incidence rates among those patients with well-controlled (≥80% time undetectable HIV viral load) and without well-controlled HIV. Univariable Poisson regression yielded incidence rate ratios with 95% confidence limits for assessing the magnitude and significance of the difference between the NSCLC rates in the two groups.

We then estimated adjusted hazard ratios (aHR) and 95% confidence intervals (Cis) for the association between potential risk factors and the risk of developing NSCLC in HIV-positive smokers using Cox proportional hazards regression models with time-varying predictors. This began with the estimation of unadjusted (univariable) regression models. Model predictors and covariates were retained in multivariable models, according to a combination of a priori (“forced in”) and posteriori (significance driven) criteria. Among those retained a priori were the demographic variables, period of HIV index and time-updated, BMI, and nadir CD4. The other factors were subjected to an initial requirement of unadjusted *p* < 0.15 for further consideration in multivariable analyses. The three lipid variables were retained or eliminated as a group. Throughout, to assess significance for categorized variables, overall model ANOVA Wald tests were used (which are not sensitive to the choice of reference levels).

In the regression models, death was treated as a censoring event, which allows for the interpretation of the model estimates as yielding cause-specific hazard ratios with death as a competing event. Analyses were performed using SAS version 9.4 (SAS Institute, Cary, NC, USA). Statistical significance was determined with two-sided Wald tests at α = 0.05.

The study protocol was approved by the Institutional Review Board at Baylor College of Medicine (Approval number: H-38727) and the Research and Development Committee at the Michael E. DeBakey Veterans Affairs Medical Center and the Puget Sound VA Healthcare System.

## 5. Conclusions

In summary, we show here that besides older age, non-Hispanic ethnicity, history of COPD and of prior pneumonia, metabolic variables, including lower BMI at HIV diagnosis, history of cachexia among individuals with well-controlled HIV, and presence of cachexia at diagnosis are risk factors for developing NSCLC in a large cohort of veterans with HIV who have a history of smoking. Prospective studies focusing on determining potential mechanisms for these results are needed to establish the clinical significance and potential causality of these findings. Finally, these data could also inform risk stratification of PWH to determine would benefit most from aggressive lung cancer screening to optimize detection of disease at an early and potentially curative stage.

## Figures and Tables

**Table 1:** Baseline and end of study characteristics of a nationwide cohort of PWH with a history of smoking (*n* = 33,351) according to degree of HIV-viral control.

Variable	Levels	Well-Controlled HIV		Total (*n* = 33,351)
		Yes	No	
		(*n* = 13,301)	(*n* = 20,050)	
		*n* (%)	*n* (%)	*n* (%)
Age at Index date, years	<50	7010 (52.7%)	12,453 (62.1%)	19,463 (58.4%)
	50–59	4418 (33.2%)	5626 (28.1%)	10,044 (30.1%)
	60+	1873 (14.1%)	1971 (9.8%)	3844 (11.5%)
Gender	Female	332 (2.5%)	536 (2.7%)	868 (2.6%)
	Male	12,969 (97.5%)	19,514 (97.3%)	32,483 (97.4%)
Race/Ethnicity	Black	6204 (46.6%)	11,552 (57.6%)	17,756 (53.2%)
	Hispanic	803 (6.0%)	1002 (5.0%)	1805 (5.4%)
	Other	127 (1.0%)	176 (0.9%)	303 (0.9%)
	Unreported	347 (2.6%)	550 (2.7%)	897 (2.7%)
	White	5820 (43.8%)	6770 (33.8%)	12,590 (37.7%)
BMI at Index date, kg/m^2^	<18.5	219 (1.6%)	366 (1.8%)	585 (1.8%)
	18.5–25	4723 (35.5%)	5418 (27.0%)	10,141 (30.4%)
	25–30	3991 (30.0%)	3855 (19.2%)	7846 (23.5%)
	30+	1798 (13.5%)	1688 (8.4%)	3486 (10.5%)
	unmeasured	2570 (19.3%)	8723 (43.5%)	11,293 (33.9%)
LDL-C at Index date, mg/dL	<100	3573 (26.9%)	4487 (22.4%)	8060 (24.2%)
	100–129	2129 (16.0%)	2383 (11.9%)	4512 (13.5%)
	130–159	1088 (8.2%)	1141 (5.7%)	2229 (6.7%)
	160+	502 (3.8%)	527 (2.6%)	1029 (3.1%)
	unmeasured	6009 (45.2%)	11,512 (57.4%)	17,521 (52.5%)
HDL-C at Index date, mg/dL	<40	4106 (30.9%)	4622 (23.1%)	8728 (26.2%)
	40–60	2712 (20.4%)	3309 (16.5%)	6021 (18.1%)
	>60	725 (5.5%)	899 (4.5%)	1624 (4.9%)
	unmeasured	5758 (43.3%)	11,220 (56.0%)	16,978 (50.9%)
Triglycerides at Index date, mg/dL	<150	4220 (31.7%)	5590 (27.9%)	9810 (29.4%)
	150+	3709 (27.9%)	3801 (19.0%)	7510 (22.5%)
	unmeasured	5372 (40.4%)	10,659 (53.2%)	16,031 (48.1%)
History of statin use at Index date	Yes	999 (7.5%)	988 (4.9%)	1987 (6.0%)
History of diabetes at Index date	Yes	780 (5.9%)	1017 (5.1%)	1797 (5.4%)
History of COPD at Index date	Yes	938 (7.1%)	1178 (5.9%)	2116 (6.3%)
HIV diagnosis date, years	<2001	5117 (38.5%)	9428 (47.0%)	14,545 (43.6%)
	2001–2009	5399 (40.6%)	6263 (31.2%)	11,662 (35.0%)
	2010+	2785 (20.9%)	4359 (21.7%)	7144 (21.4%)
Statin exposure *	Yes	6736 (50.6%)	6299 (31.4%)	13,035 (39.1%)
Nadir CD4 *	<200	5095 (38.3%)	8729 (43.5%)	13,824 (41.5%)
	200–500	5175 (38.9%)	5170 (25.8%)	10,345 (31.0%)
	>500	1782 (13.4%)	1400 (7.0%)	3182 (9.5%)
	unmeasured	1249 (9.4%)	4751 (23.7%)	6000 (18.0%)
ART (% time) *	<50%	2558 (19.2%)	9480 (47.3%)	12,038 (36.1%)
	50–80%	4327 (32.5%)	5241 (26.1%)	9568 (28.7%)
	80%+	6416 (48.2%)	2421 (12.1%)	8837 (26.5%)
Non-cannabis substance abuse *	Yes	5389 (40.5%)	9878 (49.3%)	15,267 (45.8%)
Alcohol abuse *	Yes	6418 (48.3%)	10,547 (52.6%)	16,965 (50.9%)

Abbreviations: Index, beginning of follow-up; BMI, body mass index; COPD, chronic obstructive pulmonary disease; LDL-C, low-density lipoprotein cholesterol; HDL-C, high-density lipoprotein cholesterol; PWH, people living with HIV; well-controlled, patients on current antiretroviral therapy (ART) and with undetectable VL 80% of the time. *p* < 0.0001 for all except gender, *p* = 0.55. * Through the end of the study period. *p*-Values are all < 0.0001.

**Table 2:** Univariate Cox proportional hazard models for incident non-small cell lung cancer (NSCLC) risk in PWH with a history of smoking according to the degree of HIV viral control.

Variable	Levels	All		Well-Controlled HIV			
				Yes		No	
		HR and 95% CI	*p*-Value	HR and 95% CI	*p*-Value	HR and 95% CI	*p*-Value
Age at Index date, years	<50	1.0 (reference)		1.0 (reference)		1.0 (reference)	
	50–59	3.25 (2.75–3.84)	<0.0001	2.85 (2.12–3.83)	<0.0001	3.65 (2.97–4.48)	<0.0001
	60+	4.36 (3.49–5.45)	<0.0001	4.28 (2.97–6.16)	<0.0001	5.11 (3.82–6.85)	<0.0001
Gender	F vs. M	0.82 (0.492–1.369)	0.45	0.72 (0.27–1.94)	0.52	0.84 (0.45–1.58)	0.59
Race/ethnicity	White	1.0 (reference)		1.0 (reference)		1.0 (reference)	
	Black	0.96 (0.82–1.12)	0.58	0.95 (0.73–1.23)	0.67	0.93 (0.76–1.13)	0.43
	Hispanic	0.34 (0.19–0.58)	0.0001	0.32 (0.13–0.79)	0.01	0.33 (0.16–0.67)	0.002
	Other	0.20 (0.03–1.45)	0.11	0.57 (0.08–4.09)	0.578	0.000 (0.000–Infty)	0.96
	Unreported	0.48 (0.22–1.08)	0.07	0.44 (0.11–1.79)	0.25	0.55 (0.20–1.49)	0.24
HIV diagnosis date, years	pre 2001	1.0 (reference)		1.0 (reference)		1.0 (reference)	
	2001–2009	1.19 (1.01–1.39)	0.04	1.15 (0.87–1.51)	0.33	1.27 (1.03–1.56)	0.02
	2010+	1.11 (0.82–1.51)	0.50	0.73 (0.37–1.43)	0.35	1.34 (0.94–1.91)	0.11
Cachexia *	none	1.0 (reference)		1.0 (reference)		1.0 (reference)	
	current cachexia	6.01 (4.91–7.35)	<0.0001	7.18 (5.15–9.99)	<0.0001	5.27 (4.06–6.84)	<0.0001
	recent cachexia	1.36 (0.89–2.08)	0.16	1.49 (0.72–3.06)	0.28	1.20 (0.69–2.08)	0.51
	distant past or rebounded	1.28 (1.05–1.57)	0.01	1.44 (1.04–1.98)	0.02	1.14 (0.87–1.49)	0.35
	undefined/first year after index	1.08 (0.77–1.50)	0.67	0.81 (0.32–2.04)	0.65	0.96 (0.65–1.40)	0.82
BMI at Index date, kg/m^2^	<18.5	2.06 (1.36–3.13)	0.0007	2.29 (1.16–4.53)	0.02	1.84 (1.06–3.17)	0.03
	18.5–25	1.0 (reference)		1.0 (reference)		1.0 (reference)	
	25–30	0.63 (0.51–0.78)	<0.0001	0.71 (0.51–0.97)	0.03	0.58 (0.44–0.76)	0.0002
	30+	0.47 (0.34–0.66)	<0.0001	0.53 (0.32–0.88)	0.01	0.45 (0.29–0.70)	0.0004
	missing	0.85 (0.71–1.01)	0.07	0.77 (0.54–1.09)	0.15	0.79 (0.64–0.98)	0.03
Nadir CD4 cell count, cells/mm^3^ *	<200	1.0 (reference)		1.0 (reference)		1.0 (reference)	
	200–500	0.88 (0.73–1.05)	0.15	0.85 (0.64–1.14)	0.27	0.91 (0.72–1.15)	0.42
	>500	0.81 (0.62–1.06)	0.13	0.64 (0.41–1.01)	0.05	0.94 (0.66–1.35)	0.75
	missing	1.02 (0.80–1.29)	0.88	0.92 (0.58–1.45)	0.71	0.95 (0.71–1.28)	0.73
% Time with undetectable VL *	<50%	1.0 (reference)					
	50–80%	0.94 (0.76–1.16)	0.54	---	---	---	---
	80%+	1.01 (0.85–1.20)	0.91	---	---	---	---
H/O COPD at Index date	Y/N	2.19 (1.69–2.85)	<0.0001	2.21 (1.41–3.48)	0.0006	2.19 (1.55–3.10)	<0.0001
Current HDL-C, mg/dL *	<40	1.0 (reference)		1.0 (reference)		1.0 (reference)	
	40–60	0.84 (0.69–1.01)	0.06	0.85 (0.63–1.15)	0.30	0.86 (0.67–1.09)	0.21
	>60	0.98 (0.75–1.27)	0.85	0.99 (0.66–1.50)	0.97	0.99 (0.70–1.42)	0.99
	missing	0.64 (0.52–0.79)	<0.0001	0.61 (0.40–0.91)	0.01	0.57 (0.44–0.73)	<0.0001
Current LDL-C, mg/dL *	<100	1.0 (reference)		1.0 (reference)		1.0 (reference)	
	100–129	0.90 (0.74–1.09)	0.29	0.89 (0.64–1.22)	0.46	0.89 (0.69–1.15)	0.38
	130–159	0.77 (0.58–1.02)	0.06	0.92 (0.60–1.41)	0.71	0.68 (0.46–1.01)	0.05
	160+	0.69 (0.45–1.08)	0.10	0.49 (0.22–1.13)	0.09	0.83 (0.48–1.43)	0.50
	missing	0.65 (0.53–0.79)	<0.0001	0.67 (0.46–0.99)	0.04	0.54 (0.43–0.69)	<0.0001
Current Triglycerides, mg/dL *	<150	1.0 (reference)		1.0 (reference)		1.0 (reference)	
	150+	0.67 (0.56–0.79)	<0.0001	0.77 (0.58–1.01)	0.06	0.63 (0.49–0.79)	<0.0001
	missing	0.55 (0.45–0.68)	<0.0001	0.56 (0.37–0.84)	0.005	0.48 (0.38–0.61)	<0.0001
% Time on statins*	none	1.0 (reference)		1.0 (reference)		1.0 (reference)	
	<50%	1.28 (1.05–1.55)	0.01	1.04 (0.75–1.44)	0.81	1.49 (1.16–1.92)	0.001
	50–80%	1.31 (0.97–1.77)	0.07	1.39 (0.93–2.08)	0.11	1.51 (0.95–2.42)	0.08
	80%+	1.75 (1.25–2.44)	0.001	1.64 (1.03–2.61)	0.03	2.46 (1.48–4.07)	0.0005
Current diabetes*	Y/N	1.07 (0.87–1.32)	0.52	1.17 (0.85–1.62)	0.33	1.07 (0.81–1.41)	0.64
COPD history (so far) *	Y/N	2.57 (2.19–3.00)	<0.0001	2.43 (1.87–3.17)	<0.0001	2.72 (2.22–3.32)	<0.0001
Pneumonia episodes (so far) *	0	1.0 (reference)		1.0 (reference)		1.0 (reference)	
	1	1.80 (1.49–2.17)	<0.0001	1.83 (1.34–2.49)	0.0001	1.77 (1.40–2.24)	<0.0001
	2+	2.18 (1.66–2.84)	<0.0001	2.56 (1.68–3.99)	<0.0001	2.01 (1.43–2.83)	<0.0001
% Time on metformin (from diabetes) *	no metformin	1.0 (reference)		1.0 (reference)		1.0 (reference)	
	<50%	0.88 (0.57–1.38)	0.58	0.99 (0.50–1.98)	0.99	0.82 (0.45–1.49)	0.51
	50–80%	1.32 (0.76–2.27)	0.32	1.45 (0.66–3.18)	0.35	1.12 (0.49–2.56)	0.79
	80%+	0.68 (0.32–1.43)	0.30	0.49 (0.14–1.68)	0.26	0.78 (0.27–2.22)	0.64
	no DM	0.88 (0.66–1.19)	0.41	0.82 (0.49–1.35)	0.43	0.87 (0.59–1.27)	0.46
Alcohol abuse (so far) *	Y/N	1.29 (1.11–1.51)	0.0010	1.28 (0.99–1.66)	0.06	1.28 (1.05–1.56)	0.01
Non-cannabis substance use (so far) *	Y/N	1.14 (0.98–1.33)	0.09	1.04 (0.79–1.36)	0.76	1.17 (0.96–1.42)	0.12

Abbreviations: Index, beginning of follow-up; BMI, body mass index; COPD, chronic obstructive pulmonary disease; LDL-C, low-density lipoprotein cholesterol; HDL-C high-density lipoprotein cholesterol; NSCLC, non-small cell lung cancer; PWH, people living with HIV; well-controlled, patients on current ART and with undetectable VL 80% of the time; HR, hazard ratio; CI, confidence interval. * Time-updated variables.

**Table 3:** Multivariable hazard ratios and 95% confidence intervals for the association between HIV-infection and risk of NSCLC in PWH with a history of smoking according to agree of HIV viral control.

Variable	Levels	Well-Controlled HIV			
		Yes		No	
		HR and 95% CI	*p*-Value	HR and 95% CI	*p*-Value
Age at Index date, years	<50	1.0 (reference)		1.0 (reference)	
	50–59	2.55 (1.88–3.46)	<0.0001	3.29 (2.66–4.06)	<0.0001
	60+	3.79 (2.56–5.62)	<0.0001	4.44 (3.25–6.08)	<0.0001
Race	White	1.0 (reference)		1.0 (reference)	
	Black	0.98 (0.74–1.30)	0.89	0.94 (0.76–1.16)	0.53
	Hispanic	0.35 (0.14–0.86)	0.02	0.37 (0.18–0.75)	0.006
	Other	0.83 (0.12–5.98)	0.85	0.00 (0.00–Infty)	0.96
	Unreported	0.49 (0.12–2.01)	0.32	0.56 (0.21–1.51)	0.24
Gender	F vs. M	0.89 (0.33–2.43)	0.82	0.99 (0.53–1.88)	0.98
COPD history (so far) *	Y/N	1.87 (1.42–2.47)	<0.0001	2.07 (1.68–2.56)	<0.0001
HIV diagnosis date, years	pre 2001	1.0 (reference)		1.0 (reference)	
	2001–2009	0.98 (0.73–1.31)	0.89	1.07 (0.86–1.32)	0.56
	2010+	0.54 (0.27–1.08)	0.08	0.93 (0.64–1.37)	0.72
Nadir CD4 cell count, cell/mm^3^ *	<200	1.0 (reference)		1.0 (reference)	
	200–500	0.99 (0.74–1.33)	0.95	1.02 (0.80–1.29)	0.88
	>500	0.89 (0.56–1.42)	0.61	1.17 (0.81–1.69)	0.39
BMI at Index date, kg/m^2^	<18.5	1.73 (0.87–3.44)	0.11	1.25 (0.72–2.16)	0.43
	18.5–25	1.0 (reference)		1.0 (reference)	
	25–30	0.75 (0.54–1.04)	0.08	0.61 (0.46–0.81)	0.0006
	30+	0.52 (0.31–0.86)	0.01	0.40 (0.26–0.63)	<0.0001
Cachexia *	none	1.0 (reference)		1.0 (reference)	
	current cachexia	6.34 (4.52–8.88)	<0.0001	4.42 (3.39–5.77)	<0.0001
	recent cachexia	1.37 (0.66–2.83)	0.39	1.06 (0.61–1.83)	0.84
	distant past or rebounded	1.39 (1.01–1.93)	0.04	1.07 (0.81–1.40)	0.64
	undefined/first year after index	1.04 (0.39–2.78)	0.93	1.23 (0.80–1.88)	0.34
Current LDL-C, mg/dL *	<100	1.0 (reference)		1.0 (reference)	
	100–129	1.05 (0.76–1.45)	0.78	1.01 (0.78–1.31)	0.93
	130–159	1.18 (0.77–1.82)	0.44	0.83 (0.56–1.24)	0.36
	160+	0.67 (0.29–1.53)	0.33	1.08 (0.62–1.88)	0.79
Current HDL-C, mg/dL *	<40	1.0 (reference)		1.0 (reference)	
	40–60	0.79 (0.56–1.08)	0.13	0.77 (0.59–0.99)	0.03
	> 60	0.77 (0.49–1.19)	0.24	0.76 (0.53–1.09)	0.13
Current Triglycerides, mg/dL *	<150	1.0 (reference)		1.0 (reference)	
	150+	0.80 (0.60–1.08)	0.14	0.65 (0.51–0.84)	0.0007
Pneumonia episodes (so far) *	0	1.0 (reference)		1.0 (reference)	
	1	1.41 (1.02–1.95)	0.03	1.34 (1.05–1.71)	0.02
	2+	1.64 (1.05–2.58)	0.03	1.23 (0.86–1.75)	0.25
Alcohol (so far) *	Y/N	1.22 (0.90–1.64)	0.19	1.11 (0.88–1.40)	0.36
% Time on statins *	none	1.0 (reference)		1.0 (reference)	
	<50%	0.91 (0.65–1.28)	0.58	1.23 (0.95–1.59)	0.12
	50–80%	1.14 (0.75–1.75)	0.53	1.04 (0.64–1.69)	0.86
	80%+	1.27 (0.77–2.09)	0.34	1.59 (0.94–2.69)	0.08
Non-cannabis substance use (so far) *	Y/N	0.91 (0.66–1.25)	0.56	1.01 (0.80–1.28)	0.92

Abbreviations: Index, beginning of follow-up; BMI, body mass index; COPD, chronic obstructive pulmonary disease; F, female; LDL-C, low-density lipoprotein cholesterol; HDL-C high-density lipoprotein cholesterol; M, male; NSCLC, non-small cell lung cancer; PWH, people living with HIV; well-controlled, patients on current ART and with undetectable VL 80% of the time; HR, hazard ratio; CI, confidence interval; * Time-updated variables. Hazard ratios for missing values not reported.

## Supplementary Materials

The following are available online at https://www.mdpi.com/2072-6694/12/12/3809/s1, Figure S1: Cumulative incidence curve of non-small cell lung cancers with death as a competing event in people living with HIV (PWH) with a history of smoking.
