# Supplementary material for: Effect of Body Weight and Other Metabolic Factors on Risk of Non-Small Cell Lung Cancer among Veterans with HIV and a History of Smoking
Source: Cancers (Basel). 2020 Dec 17;12(12):3809. doi: 10.3390/cancers12123809 (PMC7765814; doi:10.3390/cancers12123809)
Supplement: Supplementary file 1 [file cancers-12-03809-s001.pdf]

cancers
Supplementary Materials

Effect of Body Weight and Other Metabolic Factors on Risk of Non-Small Cell Lung Cancer among Veterans with HIV and a History of Smoking

Jose M. Garcia, Jennifer R. Kramer, Peter A. Richardson, Sarah Ahmed, Kathryn E. Royse, Donna L. White, Suchismita Raychaudhury, Elaine Chang, Christine M. Hartman, Michael J. Silverberg and Elizabeth Y. Chiao

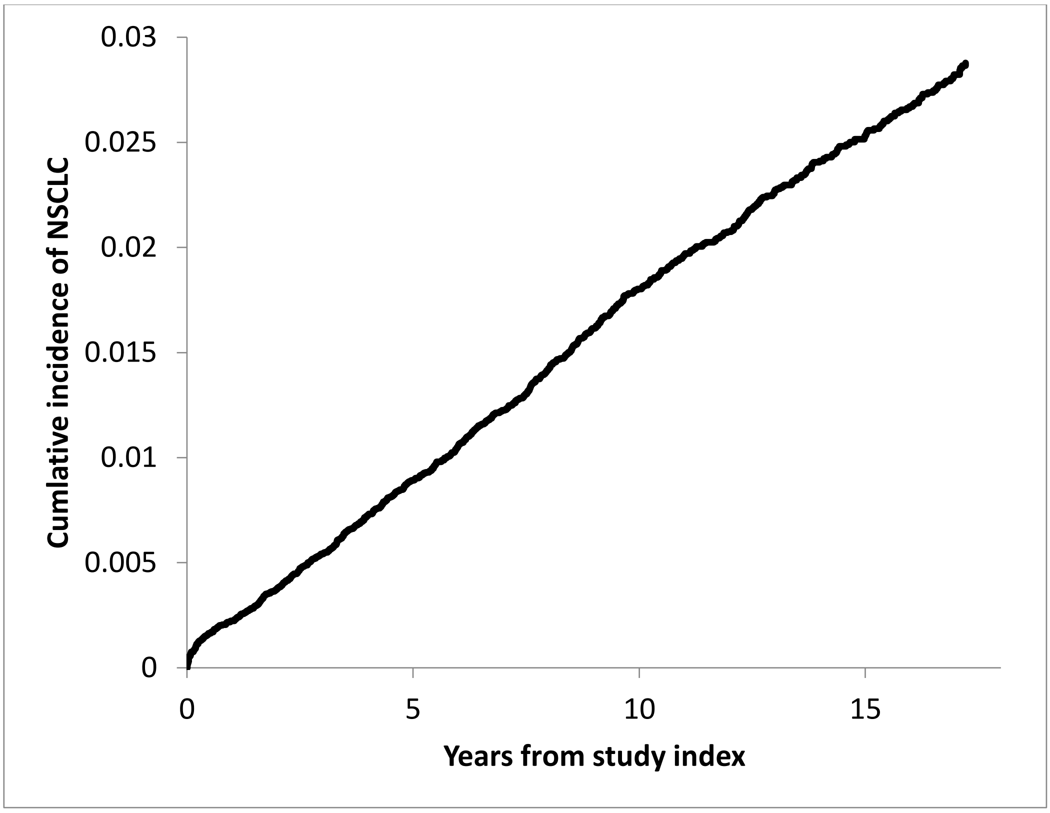

Figure S1. Cumulative incidence curve of non-small cell lung cancers with death as a competing event in people living with HIV (PWH) with a history of smoking.
